# LAP-MALDI MS coupled with machine learning: an ambient mass spectrometry approach for high-throughput diagnostics†

**DOI:** 10.1039/d1sc05171g

**Published:** 2022-01-18

**Authors:** Cristian Piras, Oliver J. Hale, Christopher K. Reynolds, A. K. (Barney) Jones, Nick Taylor, Michael Morris, Rainer Cramer

**Affiliations:** Department of Chemistry, University of Reading Whiteknights Reading RG6 6DX UK r.k.cramer@reading.ac.uk +44 (0)118 378 4550; School of Agriculture, Policy and Development, University of Reading Whiteknights Reading RG6 6EU UK; Veterinary Epidemiology and Economics Research Unit (VEERU), PAN Livestock Services Ltd, School of Agriculture, Policy and Development, University of Reading Whiteknights Reading RG6 6EU UK; Waters Corporation Stamford Avenue, Wilmslow SK9 4AX UK; Department of Health Sciences, "Magna Græcia University" of Catanzaro Campus Universitario "Salvatore Venuta" Viale Europa 88100 Catanzaro Italy

## Abstract

Large-scale population screening for early and accurate detection of disease is a key objective for future diagnostics. Ideally, diagnostic tests that achieve this goal are also cost-effective, fast and easily adaptable to new diseases with the potential of multiplexing. Mass spectrometry (MS), particularly MALDI MS profiling, has been explored for many years in disease diagnostics, most successfully in clinical microbiology but less in early detection of diseases. Here, we present liquid atmospheric pressure (LAP)-MALDI MS profiling as a rapid, large-scale and cost-effective platform for disease analysis. Using this new platform, two different types of tests exemplify its potential in early disease diagnosis and response to therapy. First, it is shown that LAP-MALDI MS profiling detects bovine mastitis two days before its clinical manifestation with a sensitivity of up to 70% and a specificity of up to 100%. This highly accurate, pre-symptomatic detection is demonstrated by using a large set of milk samples collected weekly over six months from approximately 500 dairy cows. Second, the potential of LAP-MALDI MS in antimicrobial resistance (AMR) detection is shown by employing the same mass spectrometric setup and similarly simple sample preparation as for the early detection of mastitis.

## Abstract 2

LAP-MALDI MS profiling provides rapid, cost-effective large-scale disease analysis as demonstrated by preclinical detection of bovine mastitis and antimicrobial resistance testing using a longitudinal sample collection from a 500-cows dairy herd.

## Graphical abstract

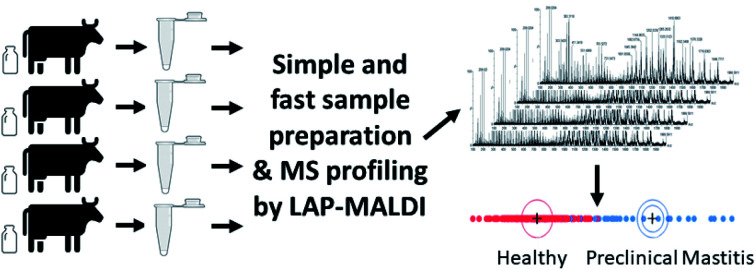

## Introduction

Infectious diseases can have devastating effects on entire populations as recently experienced with COVID-19 and predicted for future pandemics,^1^ including any compounding impact by the increasing growth of antimicrobial resistance (AMR).^2^ Recent reports and commentaries explicitly describe these effects and their consequences on health and the economy as well as potential pathways for an effective response and preventative measures to avoid or limit the impact of pandemics.^3,4^ One measure that has been frequently discussed is the use of adequate diagnostics and mass testing.^3,5^ Such discussions are typically centred around test accuracy (sensitivity and specificity), speed and capacity at low cost per sample with an adaptability of the testing platform to new diseases that will allow for rapid deployment without an overreliance on the production and sourcing of disease-specific test reagents, which could lead to supply chain issues.^6^ Ideally, such testing platform will also be capable of multiplexing, *i.e.* detecting a range of diseases, their prognosis and/or their response to therapy in one single test, and be operational within a few days, even for newly discovered diseases, using the same instrumentation as for previous disease testing.

Mass spectrometry (MS) has repeatedly been championed as a diagnostic method for highly specific and sensitive measurement of disease-specific biomarkers.^7,8^ Its exquisite mass measurement accuracy and resolution can provide the biomarker specificity and detection space for (multiplexed) disease detection at a level conventional antigen tests cannot compete.^9–11^ Particularly in combination with additional analysis modes such as MS/MS or the use of ion mobility, MS approaches can be extremely powerful with respect to detecting specific disease biomarkers unambiguously (*e.g.* see Singh *et al.*^12^).

However, most of the published MS methods are based on rather laborious and complex upfront sample separation and preparation methods as found in LC-ESI MS/MS with the additional disadvantage of demanding a minimum of several minutes of the mass analyser's time per sample. Upfront sample preparation for LC-ESI MS/MS typically involves enzymatic digestion, often leading to the loss of information related to the exact proteoform.^13^ Alternatively, MALDI MS profiling using axial linear TOF instrumentation, as established in microbiological testing, can allow for simpler upfront sample preparation and short MS analysis time but cannot provide the same high detection performance as modern atmospheric pressure (AP) ESI instruments with MS/MS capability. In this context, a major hindrance is the formation of predominately singly charged ions in (solid) MALDI while ESI generates abundant peptide/protein ions with a higher number of charges, resulting in superior fragmentation^14^ and (lower) *m*/*z* values that allow the employment of high-performing instruments with Q-TOF and orbitrap mass analysers.

Ideally, the speed and simplicity of MALDI is combined with the advantages of ESI, enabling the use of high-performing MS instrumentation. With this ideal in mind we developed a mass spectrometric platform that is based on the exploitation of liquid AP (LAP)-MALDI, which facilitates the generation of multiply charged ions and therefore the effective use of MALDI for MS(/MS) analysis on these types of instruments.^15^ In addition, the liquid nature of the MALDI sample provides an extremely stable ion yield, consuming only minute amounts of sample material (picoliters) while maintaining the speed of MALDI.^15,16^ This methodology has been further developed to include a one-pot/two-step sample preparation applicable to crude liquid biopsies, leading to an analyte solution that can be directly used for MALDI sample preparation.^17^

Here, we show the application of the above methodology for the fast and sensitive detection of pre-clinical mastitis in dairy cows using only small amounts of milk from the daily milking routine. In general, many liquid biopsies such as milk samples can be collected non-invasively and are known to harbour biomarkers that originate from pathogens and/or the host's response to infectious disease. Due to the low sample volume (μL) consumed virtually all types of mammalian milk, including human and rodent milk, are equally well suited for the analysis by this methodology.

The presented example focuses on dairy milk with its substantial impact on health and the economy. Assuming an incidence of ∼36 clinical mastitis cases per 100 cow-years and a cost of >US$200 per case and year for US dairy herds,^18^ an estimated economic loss of US$77 per cow per year was recently reported.^19^ With >100 million dairy cows world-wide (>20 million in the EU; >9 million in the USA)^20^ the economic impact amounts to billions of US dollars each year.

More importantly, antibiotic treatment of farm animals, in particular dairy cows, was also identified as a major human health risk.^21^ It has been suggested that antibiotic residues in milk can lead to allergic reactions, and zoonotic transmission of AMR strains of bacteria is a real threat and can add to AMR in humans.^21^

Thus, we also employed LAP-MALDI MS for AMR testing based on monitoring the appearance of products of antimicrobial β-lactam-containing compounds (ampicillin) as a result of bacterial lactamase activity, an approach previously applied in AMR detection by MALDI MS,^22^ using the same milk samples as for the detection of mastitis. This multi-test capacity, achieving both pre-clinical detection of mastitis and AMR testing, in combination with the method's speed and simplicity in sample preparation addresses important aspects of large-scale population screening for early and effective infectious disease intervention.

## Materials and methods

### Sample collection and biobank creation

For clinical mastitis and lactamase activity analyses, control and mastitis samples were collected by handmilking from individual quarters of the udder. Cows were assessed at each milking session (twice daily) by fore-milking each udder teat prior to attachment of the milking apparatus. If clots were present from a quarter, the cow was determined to have clinical mastitis and a milk sample was immediately collected from the affected teat. Clinical mastitis was also confirmed by the presence of redness and/or swelling of the affected gland, and in some cases elevated body temperature. Control samples were obtained randomly from cows without symptoms from one of the rear udder quarters. All samples were stored in 2 mL cryovials (Fisherbrand™, product code 10-500-26; Fisher Scientific, Loughborough, UK), placed in a dry-ice-containing polystyrene box within 5 minutes after milking and immediately stored at −80 °C after the milking session. Subsequent confirmation of infection was obtained by physical symptoms (body temperature and/or mammary swelling) and somatic cell count analysis of milk or culture to identify the infecting microorganism. Bacterial culture and assessment by (conventional) MALDI-TOF MS profiling were performed by Quality Milk Management Services Ltd (QMMS, Easton, UK).

Longitudinal milk sample collection was undertaken in 2018 over a period of 6 months (24 weeks). Around 500 individual cows were sampled weekly at the Centre for Dairy Research (CEDAR) of the University of Reading (UoR). Sampling was performed following the standard operating procedure (SOP) provided in ESI SOP 1.†

Briefly, each sample was directly taken from the sampling bottle of the 50-point rotary milking parlour (Dairymaster 50; Dairymaster (UK) Ltd, Bromsgrove, UK). Composite milk samples were obtained as a fixed proportion of the total milk collected from all four quarters of the udder during routine milking. After shaking the sampling bottle, 2 mL of the composite milk was transferred to a labelled cryovial and placed in a polystyrene box containing dry ice within 5 min of milking. After the collection of all samples for each session, the samples were placed in cryoboxes (100-Well Microtube Storage Boxes, Fisherbrand™, product code 15579811; Fisher Scientific) for long-term storage at −80 °C.

Through this continuous weekly collection procedure a dairy milk biobank was assembled that consists of approximately 12 000 samples. In addition, meta-data such as milk protein content and SCC were obtained monthly from each cow through the farm's routine milk analysis.

### Analyte extraction from milk samples

Milk samples were defrosted at room temperature and kept in an ice bath during aliquoting. For each sample, three aliquots of 20 μL were transferred to separate 96-well microtiter plates (ABgene™ SuperPlate, product code 10032013; Fisher Scientific). For model building, all clinical/pre-clinical mastitis samples were used while for the control groups samples were randomly chosen using the online list randomizer tool at http://www.random.org/lists/. Each microtiter plate had samples from all sample groups with sample groups being aliquotted and spotted row-wise but analysed column-wise as described below, thus limiting run-order, plate and time-dependent systematic errors. Two of the three aliquots were immediately stored at −80 °C in their separate plates as replicates for future analysis.

In general, aliquots in each plate were subjected to a precipitation step carried out with 100 μL of 5% (w/v) trichloroacetic acid (TCA), which was mixed with the aliquot by pipetting (dispensing/aspirating) the mixture 10 times using a 96-head CyBi™-Disk liquid handling robot (CyBio AG, Jena, Germany). Sample plates were then centrifuged for 5 min at 3000*g*. Supernatants were discarded and sample pellets were re-suspended in 80 μL of water/acetonitrile/isopropanol (1/1/1; v/v/v) by pipetting the mixtures 30 times, again using a CyBi™-Disk robot. After a sonication step of 60 s, sample plates were stored at −20 °C prior to LAP-MALDI sample preparation and MS analysis.

For analyte extraction, LAP-MALDI sample preparation and MS analysis of the longitudinally collected samples, the procedures were based on an SOP that can be found in ESI SOP 2.†

### LAP-MALDI sample preparation

Sample extracts were defrosted at room temperature for 5 min. The matrix chromophore α-cyano-4-hydroxycinnamic acid (CHCA) was dissolved to a final concentration of 30 mg mL^−1^ in acetonitrile/water (70/30; v/v) by 2 min sonication. This solution was diluted 10 : 7 with ethylene glycol by thorough mixing, resulting in the liquid support matrix (LSM) used for LAP-MALDI.

Aliquots of 5 μL of each sample extract were transferred from their storage plate to a new 96-well microtiter plate (product code 650101; GREINER BIO-ONE Ltd, Stonehouse, UK), keeping their plate positions. An equal volume of 5 μL of the LSM was added to each sample aliquot and mixed 10 times by pipetting. Droplets of 1.2 μL of the obtained sample mixtures were spotted on a 96-well Waters MALDI sample plate (Waters Corporation, Wilmslow, UK), keeping the original plate positions.

### LAP-MALDI MS analysis

**Fig. 1:**
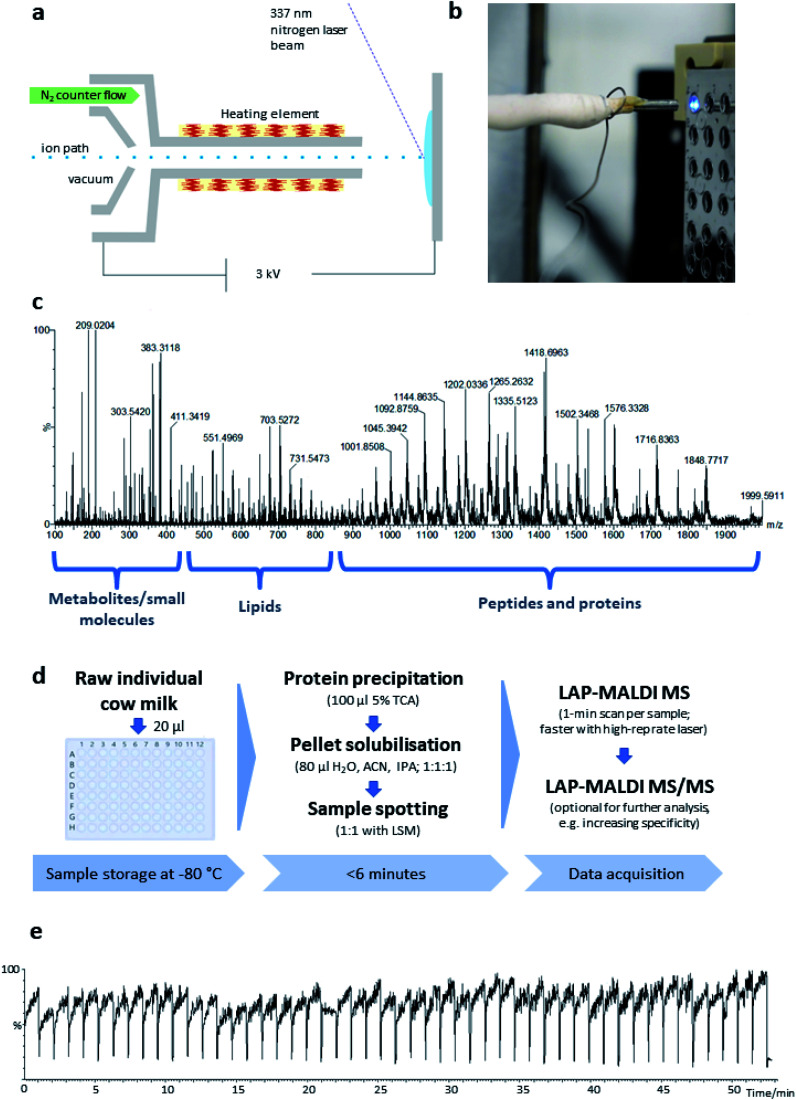
(a) Schematic of the LAP-MALDI source. (b) Close-up image of the LAP-MALDI sample irradiation, showing the ion transfer tube heated by a resistance wire opposite the sample plate with several liquid MALDI sample droplets. The irradiated sample droplet shows strong fluorescence. (c) LAP-MALDI MS spectrum (*m*/*z* 100–2000) obtained by acquiring data over one minute. (d) Workflow for the sample preparation and LAP-MALDI MS analysis of raw cow milk (TCA: trichloroacetic acid; ACN: acetonitrile; IPA: isopropanol; LSM: liquid support matrix). (e) Total ion chromatogram recorded for 50 separate milk samples analysed by LAP-MALDI MS.

LAP-MALDI MS analysis was performed on a hybrid quadrupole-time of flight mass spectrometer (Synapt G2-Si; Waters Corporation, Wilmslow, UK) with ion mobility separation. The LAP-MALDI source was a home-made modification of a Waters electrospray ion source (Waters Corporation). It was built with a heated ion transfer capillary (>200 °C) for improved MALDI plume desolvation (see Fig. 1). The applied voltage to the MALDI plate was 3 kV. Through the instrument's control software MassLynx™ (Waters Corporation) the source temperature was set to 80 °C and the cone voltage to 40 V. The samples were irradiated with a nitrogen laser (337 nm; MNL 103 LD; LTB Lasertechnik GmbH, Berlin, Germany) with a pulse energy of 20 μJ per shot focused to a diameter of approximately 100–150 μm with a pulse repetition rate of 20 Hz. Waters Research Enabled Software (WREnS) was used to control the sample plate holder's XY-stage motors (Zaber Technologies Inc., Vancouver, Canada) to move between samples.

The instrument was employed in sensitivity, ion mobility-TOF mode. Ion mobility wave velocity was set at 650 m s^−1^ with a 40 V height and a nitrogen flow of 90 mL min^−1^. The complete set of ion mobility separation and MS parameters can be found in the “_extern.inf” files recorded by the instrument's software in each raw data folder. These files are available in the University of Reading's Research Data Archive entry that is associated with this project (https://researchdata.reading.ac.uk/id/eprint/279). The MS scan rate was set to 1 Hz for combined lipids and proteins analysis. Positive ions were recorded over an *m*/*z* range of 100–2000. External TOF calibration was performed manually by AP-LDI MS as previously described,^23^ using sodium iodide, an acquisition time of 3 min with an *m*/*z* range of 100–2000 and Intellistart software (MassLynx™; Waters). All samples were analysed with an acquisition time of 1 min. For large-scale whole-plate analyses, samples were analysed column-wise, thus crossing several times during a whole-plate MS profiling run the various sample groups that were spotted row-wise.

### Lactamase activity analyses

For the evaluation of endogenous lactamase activity, aliquots of 20 μL of defrosted milk from the clinical mastitis study were transferred into a 96-wells microtiter plate (ABgene™ SuperPlate). After the transfer, the aliquots were gently vortexed and incubated at 37 °C for 90 min for bacterial revitalisation. Ampicillin was then spiked into every sample to a final concentration of 100 μg mL^−1^. Subsequently, all samples were gently vortexed and incubated for 2 h at 37 °C. After incubation a volume of 80 μL of methanol was added to every sample. Samples were thoroughly vortexed and centrifuged at 3000*g* for 5 min. The supernatant was analysed by LAP-MALDI MS using the same LAP-MALDI sample preparation and instrumental setup as described above. For calibration, penicillinase from *Bacillus cereus* (product code P0389; Sigma-Aldrich, Gillingham, UK) was spiked before the incubation step into control milk (*i.e.* milk without endogenous lactamase activity) at the concentrations detailed in the results description. LAP-MALDI MS ion signal intensity ratios of the protonated decarboxylated hydrolysed ampicillin (*m*/*z* 324.14) and the protonated intact ampicillin (*m*/*z* 350.12) were calculated for further analysis.

For the initial LAP-MALDI MS analysis (*m*/*z* 300–400) of milk compounds, ampicillin spiked into milk and lactamase-induced ampicillin products, a volume of 20 μL of pooled milk was used from 100 control milk samples of the pre-clinical mastitis sample collection that were defrosted for 2 h at room temperature. Some aliquots of the pooled milk were prepared as described above while others were prepared as described above without incubation with penicillinase or as described above without both ampicillin and incubation with penicillinase.

### Data processing/analysis

For classification analysis, raw MS datasets were processed with Abstract Model Builder (AMX; version 1.0.1563.0; Waters). For each sample, the AMX-processed LAP-MALDI mass spectral profile was obtained by summing all MS scans within the ‘retention’ time when that sample was analysed, discarding the ion mobility drift time information as this information was not needed for classification analysis. The final ion intensity data matrices were obtained by binning the mass spectral intensities every 1 Thomson, normalised relative to the sum of the intensities of all bins and saved as .csv files, thus obtaining datasets that are well served by the mass resolving power (>10 000) and measurement accuracy (<10 ppm) that many modern mass spectrometers can easily provide. These .csv files were subsequently analysed with JMP software (version 14; SAS Institute Inc., Marlow, UK) using the discriminant analysis (Wide Linear) function.

A training set of LAP-MALDI mass spectral profiles was used for every machine learning model generated. A separate set of LAP-MALDI mass spectral profiles was then used to test these models. Both data sets were obtained by the same mass spectral acquisition (see LAP-MALDI MS analysis), using the same sample preparation (see LAP-MALDI sample preparation). After all LAP-MALDI mass spectral profiles were acquired, an appropriately blinded classification analysis was achieved by applying fully randomised or randomised representative sampling for the training and test sets.

## Results and discussion

### LAP-MALDI source design and performance

The LAP-MALDI source is designed for use with high-performing mass spectrometers enabling the detection of heterogeneous analyte ions over a wide mass range, *i.e.* metabolites and other small molecules, lipids and peptides/proteins. Fig. 1 illustrates the ion source set-up (Fig. 1a and b) and the type of data that can be obtained from liquid biopsies such as milk as prepared for this study (Fig. 1c–e).

In Fig. 1c, a mass spectrum of dairy milk is shown that was acquired on a modern Q-TOF mass analyser in less than 1 min by LAP-MALDI MS, employing an equally fast and simple sample preparation protocol (Fig. 1d), which only requires two steps and can be undertaken in the same vial prior to MALDI sample spotting. The *m*/*z* region of 250–450 is mostly populated by sugars, metabolites and other small endogenous and exogenous compounds such as antibiotics and their products (see below). Lipids can be typically found in the *m*/*z* region of 450–850. These two areas of the spectrum are mainly dominated by singly charged [M + H]^+^, [M + Na]^+^ or [M + K]^+^ ion species. The region above *m*/*z* 800 is mostly populated by multiply charged peptides and proteins, which is one of the unique features of LAP-MALDI. The LAP-MALDI source also offers rapid movement between samples. If the laser continuously fires, the total ion current (TIC) is elevated when the laser beam hits the samples but is reduced to baseline-level intensities when the laser beam hits the blank sample plate between samples. Fig. 1e displays the TIC obtained by rapidly moving between samples and acquiring data from a central spot of each liquid MALDI sample droplet for approximately 1 min with a continuous laser pulse repetition rate of 20 Hz. The drop in TIC between samples (see Fig. 1e) conveniently separates the acquired data of each sample.

### Identification of lipids and proteins in LAP-MALDI MS profiles

**Fig. 2:**
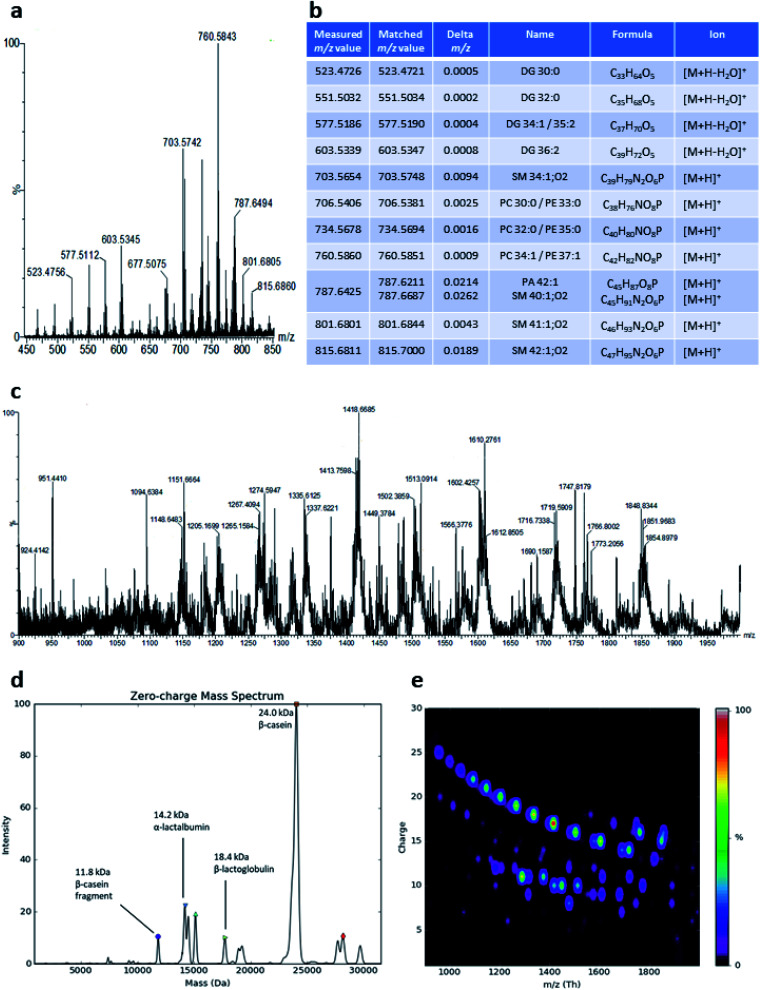
(a) LAP-MALDI mass spectrum of dairy milk, displaying the *m*/*z* range of 450–850. (b) Putative identification details for the most abundant lipid species detected in the mass spectrum shown in panel a (based on mass accuracy, literature and occurrence in the MCDB database at mcdb.ca). (c) LAP-MALDI mass spectrum of dairy milk, displaying the *m*/*z* range of 900–2000, where most proteins and peptides are detected as multiply charged ions. (d) Zero-charge mass spectrum obtained from the deconvolution of the mass spectrum shown in panel c. (e) *m*/*z-vs.*-charge ion intensity signal plot showing the charge train distributions for the ion signals of the mass spectrum shown in panel c.

LAP-MALDI MS profiles as shown in Fig. 1c include the presence of lipids, mainly as [M + H]^+^ ions of the intact lipids or after the loss of water (Fig. 2a and b). The most abundant ions detected with this method originate from diacylglycerols, phosphocholines, triacylglycerols and sphingomyelins (Fig. 2b). LAP-MALDI MS also allows for the efficient ionisation and detection of proteins and protein fragments as multiply charged ion species.

The set-up used for this study allowed data acquisition with a laser pulse repetition rate of 20 Hz and the acquisition of informative MS profiles within 1 min, switching between samples in just 2 s and without any detectable carry-over. Fig. 2c displays the richness of the proteoforms obtained in the LAP-MALDI MS profiles in the *m*/*z* range of 900–2000. Deconvolution of the spectrum in Fig. 2c reveals abundant milk proteoforms, including caseins, lactalbumins, and lactoglobulins (Fig. 2d). Larger proteins are detected with higher charge states, essentially to the same extent as in ESI† (*cf.*Fig. 2e). For β-casein (∼24 kDa), ions are detected with 15–25 charges. Ion signal trains of these proteins can be easily observed in charge-*vs.-m*/*z* plots (Fig. 2e).

### Detection of clinical and pre-clinical bovine mastitis

Samples were collected at the Centre for Dairy Research (CEDAR) at the University of Reading, UK, from a dairy herd of approximately 500 cows that are routinely milked twice daily on a 50-point rotary milking parlour. A milk sample from each cow is monthly analysed for somatic cell count (SCC), milk volume, protein content and fat content for routine herd recording (see ESI Table S1†). Cases of clinical mastitis are determined by the farm personnel through the detection of visible signs in milk (changes in colour, presence of clots) and changes of the mammary gland (swelling, abnormal tissue colour) and overall animal condition (body temperature, feed intake). LAP-MALDI MS profiles were acquired over the *m*/*z* range of 100–2000. The entire *m*/*z* range was used for classification analysis.

**Fig. 3:**
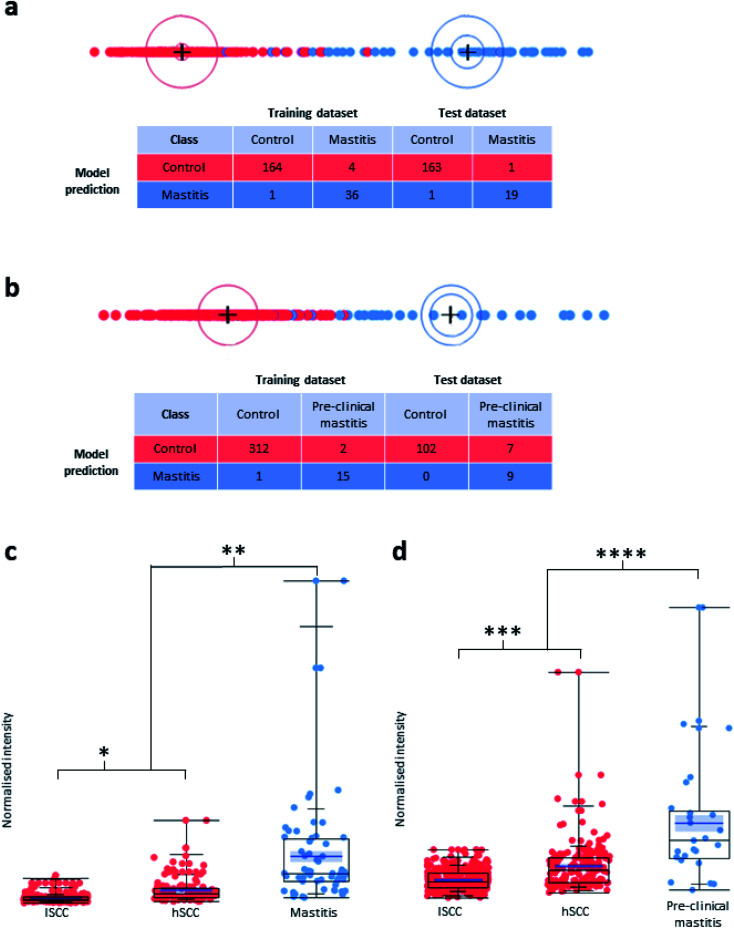
(a) Graphic representation (biplot) of the LDA model and the confusion matrix for its prediction of clinical mastitis. (b) Graphic representation (biplot) of the LDA model and the confusion matrix for its prediction of pre-clinical mastitis. The observations and the multivariate means of each group are represented as points on the biplot. They are expressed in terms of the first two canonical variables. The point corresponding to each multivariate mean is denoted by a plus (“+”) marker. A 95% confidence level circle is plotted for each mean. If two groups differ significantly, the confidence circles tend not to intersect. A circle denoting a 50% contour is also plotted for each group. This depicts a region in the space of the first two canonical variables that contains approximately 50% of the observations, assuming normality. (c and d). Normalised signal intensities of the AMX mass bin at *m*/*z* 724.5, which contains the [M + 6H]^6+^ ion species of isracidin-like peptide, using the data from the classification of clinical (c) and pre-clinical (d) mastitis. * *p*-value: 8.04 × 10^−18^; ** *p*-value: 4.84 × 10^−34^, fold change 5.81; *** *p*-value: 3.43 × 10^−17^; **** *p*-value: 4.09 × 10^−26^, fold change: 2.12. See text for more details.

For detecting clinical mastitis, a total of 60 clinical mastitis samples and 329 control samples were prepared for LAP-MALDI MS analysis (see Methods section for details). The control samples included 223 low-SCC (lSCC; defined as <200 000 somatic cells per mL of milk) samples and 106 high-SCC (hSCC; defined as ≥200 000 somatic cells per mL of milk) samples. Both lSCC and hSCC samples were used for the control group. Using a training set for multivariate machine learning, a prediction model was obtained and applied to the LAP-MALDI MS data from a test set. Details about the make-up of the control group as well as the training and test sets can be found in ESI data 1.† The analysis of the test set allowed the detection of clinical mastitis with a classification accuracy of 98.9% (Fig. 3a).

For the investigation of pre-clinical mastitis, milk sample aliquots (20 μL) were taken from a biobank of around 12 000 milk samples that were collected weekly on the same day for a period of 24 weeks (July 2018–December 2018). The collection date was compared to the date of clinical mastitis detection. From this longitudinal sample collection a total of 500 control samples and 90 mastitis samples that were collected between 0 and 7 days before the clinical event were analysed. As before, both lSCC and hSCC samples were used for the control group, and various training sets based on the days before mastitis was clinically diagnosed were subjected to multivariate machine learning to build prediction models for early (pre-clinical) detection of mastitis. Details about the make-up of the control group as well as the training and test sets used for the data in Fig. 3 can be found in ESI data 1.†

The prediction models that included mastitis samples collected more than 3 days before clinical diagnosis had a sensitivity of less than 50%. There was a substantial increase in sensitivity for models that were built using mastitis samples that were collected within 2–3 days before mastitis was clinically detected. For instance, the model that was built using the mastitis cases collected 0 and 1 day before the clinical event showed a specificity of 100% and sensitivity of 56.3% for the prediction of pre-clinical cases from analysis of samples obtained 2 days before the clinical event (Fig. 3b). The associated Volcano plot of its classification data is provided in ESI Fig. S1.† Other analyses using training and test sets that were mixtures of mastitis samples collected at various time points up to 2 days before the clinical event showed an even higher sensitivity of up to 70%.

In both LDA models of Fig. 3, the most important feature for mastitis detection is the isracidin-containing peptide ion, an α-s_1_-casein fragment, whose [M + 6H]^6+^ monoisotopic ion signal was detected at *m*/*z* 723.9 with a measurement accuracy of <5 ppm for most profiles (see ESI Fig. S2†). The majority of its isotopologues' ion signal can be found in the AMX mass bin at *m*/*z* 724.5 (see Methods section for data processing details). The box plots for the normalised signal intensity of its mass bin for the various sample sets show that the lSCC control sample set is well separated from the mastitis samples for the detection of both clinical and pre-clinical mastitis with respect to the third quartile of the lSCC control samples and first quartile of the mastitis samples (see Fig. 3c and d). Furthermore, the normalised mass bin signal intensity was significantly different between the clinical/pre-clinical mastitis sample group and the two sub-groups (lSCC, hSCC) of the control sample group (see Fig. 3c and d).

The *m*/*z* bins of 543.5, 620.5, 724.5, and 868.5/869.5, which can be assigned to the isracidin-containing peptide ions with a charge state of 5–8, have all been found with significantly differential signal intensities between pre-clinical mastitis and control samples with a change of >20% (>100% for 724.5; see ESI Fig. S1†). Other *m*/*z* bins that show significantly different signal intensities between pre-clinical mastitis and control samples are the *m*/*z* bins 728.5 and 784.5–788.5, which can be putatively assigned to another α-s_1_-casein fragment with the amino acid residues R_1_-F_24_ and a C-terminal fragment of β-casein with the amino acid residues T_126_–V_209_. Furthermore, the *m*/*z* bins 753.5/754.5 and 811.5–814.5 also fall into the same category and cover the *m*/*z* values for the 14^+^ and 13^+^ ions of a previously identified clinical mastitis marker.^17^

### Analysis of small biomolecules

**Fig. 4:**
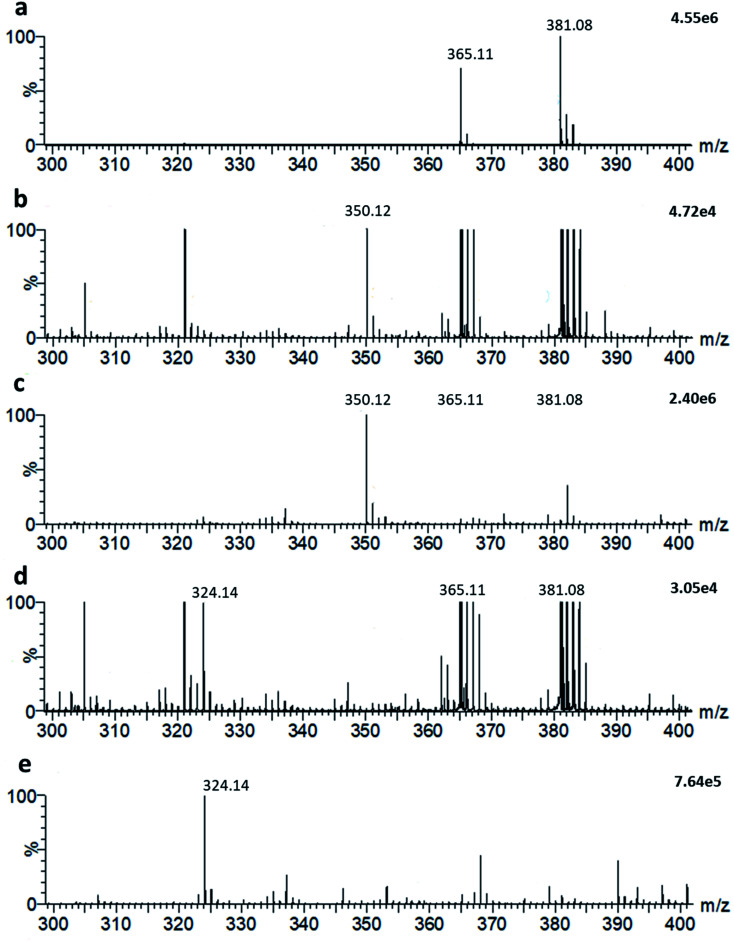
(a) LAP-MALDI mass spectrum of bovine milk (*m*/*z* 300–400). Sodiated lactose [M + Na]^+^ is detected at *m*/*z* 365.11 and potassiated lactose [M + K]^+^ at *m*/*z* 381.08. (b) LAP-MALDI mass spectrum of ampicillin (*m*/*z* 350.12; [M + H]^+^), spiked into milk at a concentration of 50 μg mL^−1^ (c) LAP-MALDI mass spectrum of ampicillin (*m*/*z* 350.12; [M + H]^+^) in water at a concentration of 50 μg mL^−1^. (d) LAP-MALDI mass spectrum of decarboxylated hydrolysed ampicillin (*m*/*z* 324.14; [M + H]^+^), obtained after 120 min incubation of ampicillin in milk (50 μg mL^−1^) with 3–6 units of penicillinase. (e) LAP-MALDI mass spectrum of decarboxylated hydrolysed ampicillin (*m*/*z* 324.14; [M + H]^+^), obtained after 120 min incubation of ampicillin in water (50 μg mL^−1^) with 3–6 units of penicillinase.

It is possible to detect simultaneously metabolites and other small molecules within the same LAP-MALDI MS profile that is used for the detection of peptides and proteins. For example, endogenous lactose is one of the dominating small molecule species when analysing milk and, as shown in Fig. 4a, its sodiated ion [M + Na]^+^ and potassiated ion [M + K]^+^ are easily detected at *m*/*z* 365.1060 (theoretical: 365.1054) and *m*/*z* 381.0812 (theoretical: 381.0794), respectively. The exogenous antibiotic compound ampicillin can also be detected by LAP-MALDI MS profiling when spiked into milk at concentrations appropriate for assaying lactamase activity (Fig. 4b). In comparison to water (Fig. 4c), the detection of ampicillin at these concentrations was not substantially impaired by the milk biomatrix despite the high background of lactose ion signals (Fig. 4b). Similarly, there was no substantial difference between milk and water in detecting decarboxylated hydrolysed ampicillin after penicillinase treatment (see Fig. 4d and e).

### Detection of ampicillin-resistant bacteria in milk

**Fig. 5:**
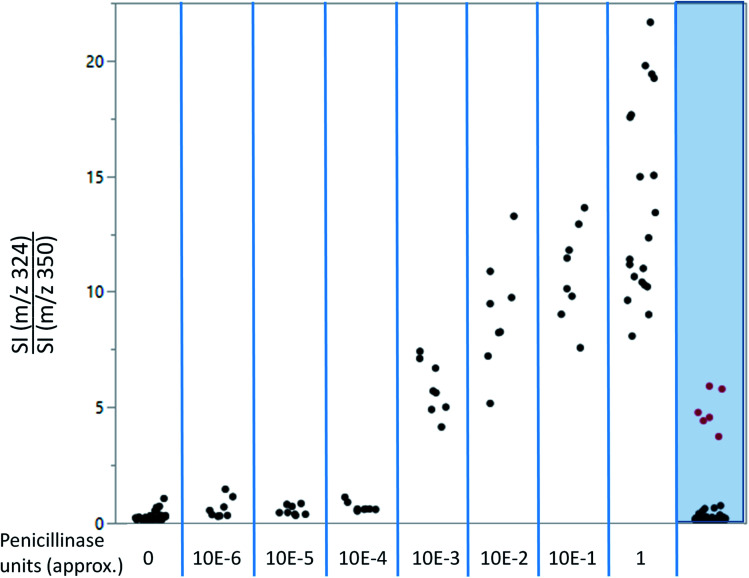
LAP-MALDI MS signal intensity ratio of the protonated decarboxylated hydrolysed ampicillin (*m*/*z* 324.14) and the protonated intact ampicillin (*m*/*z* 350.12) for various amounts of penicillinase spiked into raw healthy milk (first 8 columns/sample groups) and for clinical mastitis samples (last column/sample group). The clinical mastitis group consisted of 60 samples of raw mastitis milk analysed in duplicate. Each dot represents one technical replicate. Both duplicates of three samples (in red) show elevated levels of lactamase activity, clearly separating them from the bulk of the other mastitis cases. Routine bacteriological testing confirmed that one of the 3 samples contained *E. coli* bacteria resistant to ampicillin. The other two samples were not tested by routine bacteriological testing. Bacteriological testing of dairy milk is typically bacterium-specific and not all bacteria are routinely tested for AMR. Thus, the other two samples identified by LAP-MALDI with elevated lactamase activity can fill this gap and show the potential for multiplex testing of AMR. SI: signal intensity.

As shown in Fig. 4, ampicillin and its lactamase-generated product are easily detected when both ampicillin and lactamase are added to milk. To explore whether lactamase activity from antimicrobial resistant strains of bacteria can be detected in milk collected from mastitic cows, small aliquots (20 μL) of the previously collected 60 milk samples from symptomatic cows were incubated with ampicillin (100 μg mL^−1^) for 2 hours at 37 °C. By calculating the LAP-MALDI MS signal intensity ratio of the ion peak at *m*/*z* 324.12 (protonated decarboxylated hydrolysed ampicillin) and *m*/*z* 350.14 (protonated ampicillin), the duplicates of 3 samples, *i.e.* 6 LAP-MALDI MS profiles, showed substantially elevated ratios of ≥3 compared to all other samples, for which all duplicates resulted in ratio levels of <1 (Fig. 5). A comparison with the ratio levels obtained from milk of non-mastitic cows, which was spiked with various amounts of penicillinase, indicates that the lactamase activity in these three samples was equivalent to around 10^−3^ units of penicillinase (see Fig. 5).

### Discussion and future applications

Amongst recent improvements in ambient ionisation there have been advances in MALDI MS profiling, using liquid MALDI matrices and high-performing orthogonal mass analysers with a heated ion transfer tube.^15,17,24,25^ These advances have led to the exploitation of the combined strengths of MALDI MS profiling (speed and simplicity), as known from clinical microbial biotyping using axial vacuum TOF analysers, and the unique capability of liquid AP-MALDI to generate multiply charged peptide/protein ions. Multiply charged ions with their lower *m*/*z* values allow the use of high-performing hybrid mass analysers, as typically used with ESI, and generally provide superior MS/MS data for the structural elucidation/sequencing of larger biomolecules such as peptides and proteins.^14^ The latter was not exploited in this study since the (quantitative) ion signal differences of the MS profile's host-derived biomarkers, and not the (qualitative) identification/sequencing of specific pathogen-derived biomarkers, are the key aspect in rapid and accurate detection of diseases that are induced by multiple pathogens. However, sequencing of pathogen-derived peptides/proteins will be an important future strength of LAP-MALDI MS.

Since low *m*/*z* values for all analytes enabled the use of a high-performing orthogonal (hybrid) mass analyser, important advantages such as the simultaneous detection of the entire biomarker panel (metabolites, lipids and peptides/proteins) were achieved. This uniquely widened the range of simultaneously accessible biomarker signals for MALDI MS profiling analysis. Conventional solid MALDI with its predominately singly charged ions typically fails in detecting proteins and large peptides on these instruments since the *m*/*z* values are too high.^26^

These new functionalities gained through LAP-MALDI were explored on a commercial Q-TOF mass spectrometer, whose standard ESI ion source was simply modified by adding a heated transfer tube and MALDI stage.^25,27,28^ The milk spectra which were obtained from a fast and simple one-pot/two-step sample preparation (see Fig. 1) are extremely rich in biomarkers across a wide mass range, from metabolites and lipids to peptides and proteins, with excellent signal-to-noise ratios and high mass accuracy and resolution, as typically recorded for ESI-Q-TOF MS. The simple and fast (off-line) MALDI sample preparation as well as swift sample introduction are however unrivalled by ESI while conventional solid MALDI MS as found in clinical biotyping^29^ would not be able to detect simultaneously the full profile (set of biomarkers) due to the limitations of axial TOF mass analysers. Signal stability, which is important for profile comparison, and the added speed advantage by not loading the sample plate into a vacuum chamber are additional advantages of LAP-MALDI.

Initial tests of LAP-MALDI for the detection of clinical mastitis revealed an extremely high test accuracy of 98.9% with only two false test results. Importantly, the false positive was an hSCC sample, which is not too surprising, considering that hSCC is frequently used as a marker for mastitis.^30–32^ The fact that the percentage of hSCC samples in the test set was higher than what is typically found in the entire herd might have further increased the chance to obtain a false positive.

Interestingly, results from both the clinical and pre-clinical mastitis detection analyses reveal that the ion signal of a single, isracidin-containing peptide is significantly different between the lSCC, hSCC and mastitis samples. Single molecular markers can be useful for the design of non-MS tests such as antigen-based lateral flow tests for point-of-care testing. The <5 ppm mass measurement accuracy achieved in this study is unique for MS analysis of large peptides from a crude biofluid using only a simple and quick analyte extraction. The high mass resolving power of >10 000 throughout the entire *m*/*z* range is rarely reported for MALDI MS profiling of biofluids and substantially superior compared to analyses on axial MALDI-TOF instruments as currently employed in clinical laboratories, even when optimised for a specific *m*/*z* range.^33^ In combination with MS/MS, this analytical performance supports effective multiple reaction monitoring (MRM) of both small molecules and (large) peptides/proteins, and thus introduces entirely new diagnostic opportunities for MALDI-based platforms.

The isracidin-containing peptide was also found as highly discriminative in an earlier study on MS-based mastitis detection.^17^ Isracidin has been frequently reported as an antimicrobial peptide (AMP)^34^ but it is only more recently that the isracidin-containing peptide as found in this study has also been identified as AMP,^35^ displaying similar antimicrobial activity as isracidin. It should be noted that reported antimicrobial activity is typically determined *in vitro* against specific pathogens (bacterial strains) and can therefore not be compared to direct host response data as obtained here. However, as can be seen from the data in Fig. 3c and d, the ion signal of this peptide on its own is an insufficient biomarker for high-accuracy mastitis testing. Our data show that the entire LAP-MALDI MS profile is far more powerful than individual biomarker signals, which is further supported by the Volcano plots obtained from the MS profiles.

It is worth noting that the possibility to perform top-down MS analysis of proteins,^36^ directly from the samples used for disease detection by MS profiling, allows greater characterisation of biomarkers compared to bottom-up approaches using enzymatic digestion as used in LC-ESI MS/MS. In the study presented here, the superior top-down analysis afforded by LAP-MALDI is exemplified by the identification of a casein peptide as one of the most valuable diagnostic markers, containing isracidin and an additional 14 amino acids. The peptide's low prominence in the literature is arguably also due to the limitations of bottom-up proteomics, which generally impedes the identification and quantification of specific proteoforms.^13^ This kind of knowledge gain could warrant new peptide validations with respect to enhanced antimicrobial activity.

To test LAP-MALDI MS profiling for early disease detection, a longitudinal bovine mastitis study was initiated using simple machine learning models. The data from this study resulted in an overall sensitivity of up to 70% for the detection of mastitis 2 days before its clinical manifestation. In all tested models, sensitivity was greater than 50% and specificity was up to 100%. From model building, it was evident that the sensitivity of detecting mastitis 3 or more days in advance was well below 50%. Please note that this rapid decline in sensitivity was expected as numerous *in vivo* challenge studies have shown that inoculation of even small amounts of bacteria leads to clinical symptoms within 1–2 days.^30,31,37^ Cytokine and other host protein levels showed a similar, in some cases even earlier, temporal response.^30,32,38^ Thus, it is virtually impossible to predict individual bovine mastitis more than 2–3 days before symptoms occur unless the infection event itself can be predicted.

Given the above test performance, daily testing using LAP-MALDI MS profiling should enable the detection of at least half of the pre-symptomatic cases. The test's high specificity would only produce a few, if any, false positives and can therefore support effective mastitis control by early pharmacological (antibiotic, steroidal and non-steroidal drugs) and/or non-pharmacological (such as isolation and separate milking) intervention, dramatically reducing transmission of infection and number of cases in the herd.

Importantly, the test is also simple, fast and cost-effective, using only a microcentrifuge tube (or microtiter plate at scale-up), a couple of tips (∼US$0.02 per sample) and minute amounts of inexpensive solvents/reagents (≤US$0.01 per sample). At large scale, it is therefore significantly less costly than current biomolecular methods for bovine mastitis detection. Moreover, data acquisition time can be significantly shortened by at least a factor of 10, using a laser with a faster pulse repetition rate without losing any ion signal intensity,^39^ and a throughput of 5–10 million samples per year per platform is feasible. The capital cost of an adequate Q-TOF mass spectrometer and sample preparation robotics (≤US$300 000) and the associated running costs (including staff) would add another ≤US$0.02 per sample based on 5 years of depreciation, leading to an estimated overall cost per sample of ≤US$0.067 (for milking parlours that can automatically collect small amounts of milk samples). ESI Table S2† provides a detailed costing schedule. Interestingly, the highest contributions to the cost per sample originate from the plastic ware (tips, microtiter plates) while the instrument capital and running expenditures as well as the staff and transportation expenditures together only add around a quarter to the overall cost per sample. In summary, daily testing would cost approximately US$24 per cow per year, *i.e.* less than a third of the estimated cost of mastitis of US$77 per cow per year.^19^ Most of the costing is intrinsically the same for the analysis of milk from other mammals, including human milk analysis.

Other important outcomes of early detection and intervention of microbial infections are a reduction of antibiotic usage and improved health and welfare. For bovine mastitis, these additional benefits are well aligned with the one health concept.^40^ As the use of antibiotics in farm animals has been linked to an increase of AMR bacterial strains in humans,^41^ any reduction of antibiotics on farms and a generally healthier livestock would also benefit human health, helping in the fight of more pandemics and the global threat of AMR.

With respect to AMR the data from this study also show that LAP-MALDI MS profiling can effectively detect lactamase activity from a simple mass spectral read-out, using crude milk spiked with a conventional beta-lactam antibiotic. Using the same MS set-up as for mastitis detection, lactamase-based AMR can be detected after a short incubation period (2 hours or less) and a sample preparation that is even simpler and faster than that for mastitis detection, acquiring the mass spectral data within seconds. This workflow substantially cuts the time to detect AMR compared to classical bacteriological AMR testing, which typically involves the growth of individual bacterial colonies, potentially missing AMR strains.

In general, incubating crude liquid biopsies such as raw milk with the antibiotic of interest will provide direct and universal detection of AMR and is easily extended to other antibiotic substrates either within the same vial or if needed in different aliquots, without a substantial increase in time or cost.

Applications of this additional mode of analysis are countless in both animal and human diagnostics and can be employed beyond bacterial infections in milk. As the screening is extremely rapid and can be multiplexed with a variety of antibiotics it will certainly save time and cost compared to individual, non-exhaustive bacteriological tests as currently applied to milk samples. Importantly, due to the variety of antibiotics that can be tested at the same time, it is far more likely to identify in a much shorter time frame the antibiotics that are still effective, which can then be administered at an earlier disease stage, thus accelerating the efficient treatment and ultimately healing process as well as reducing the risk of further AMR proliferation.

It is noteworthy that simple LAP-MALDI MS profiling was sufficient for this assay despite the high biomatrix background that crude biofluids such as milk provide. Thus, other liquid biopsies such as blood will also benefit from the robustness of LAP-MALDI MS profiling. Moreover, LAP-MALDI used on high-performing MS/MS analysers also provides the opportunity to employ MRM-like assays, providing additional analytical sensitivity and specificity, if needed. However, the most important proteinaceous classifier markers obtained in this study are mostly abundant (host response) proteins or protein fragments, although the diversity in analyte types and the number of individual analyte ions detected appear to be greater compared to conventional solid MALDI MS profiling. The latter is mainly due to the relatively low background ion signals of MALDI matrix, biomatrix and other non-specific (contaminant) compounds in LAP-MALDI MS as employed in this study.

Finally, it is reiterated that future tests based on LAP-MALDI MS profiling are envisaged to be applied to other biofluids, exploiting the MS/MS capabilities provided by multiply charged ions and the use of high-performing tandem mass spectrometers, *e.g.* for pathogen identification by MS/MS sequencing and superior disease detection by MRM of pathogen and/or host peptides. Combined with the high speed of analysis, low cost per sample and multiplexing capability as well as the potential to adapt the predictive read-out extremely fast to new infectious diseases, these platforms are an appealing proposition for highly specific, large-scale population screening not only of farm animals but other species, including the entire human population.

## Data availability

Data supporting the results reported in this paper are openly available from the University of Reading Research Data Archive at https://doi.org/10.17864/1947.000279.

## Author contributions

Writing – original draft, data curation, formal analysis, project administration, visualization, investigation: CP and RC; methodology: CP, CKR, AKJ, NT, MM, and RC; resources: CKR, AKJ, MM, and RC; funding acquisition: MM and RC; conceptualization: RC; supervision: CKR, MM, and RC; validation, writing – review & editing: all authors.

## Conflicts of interest

There are no conflicts to declare.

## Abbreviations

AMP: Antimicrobial peptide
AMR: Antimicrobial resistance
AP: Atmospheric pressure
ESI: Electrospray ionisation
HPLC: High-performance liquid chromatography
LAP: Liquid AP
LC: Liquid chromatography
LDA: Linear discriminant analysis
LOD: Limit of detection
MALDI: Matrix-assisted laser desorption/ionisation
MRM: Multiple reaction monitoring
MS: Mass spectrometry
MS/MS: Tandem MS
PCR: Polymerase chain reaction
TCA: Trichloroacetic acid
TIC: Total ion current
WREnS: Waters research enabled software

## Supplementary Material

SC-013-D1SC05171G-s001

SC-013-D1SC05171G-s002

SC-013-D1SC05171G-s003

SC-013-D1SC05171G-s004

SC-013-D1SC05171G-s005

SC-013-D1SC05171G-s006
