# Supplementary material for: LAP-MALDI MS coupled with machine learning: an ambient mass spectrometry approach for high-throughput diagnostics
Source: Chem Sci. 2022 Jan 18;13(6):1746–58. doi: 10.1039/d1sc05171g (PMC8826629; doi:10.1039/d1sc05171g)
Supplement: SC-013-D1SC05171G-s001 [file SC-013-D1SC05171G-s001.pdf]

Electronic Supplementary Material (ESI) for Chemical Science.
This journal is © The Royal Society of Chemistry 2022

Supplementary Data 1: Details regarding the reported prediction models

Clinical Mastitis detection

For detecting clinical mastitis by LAP-MALDI MS, milk samples (~2 mL) were collected by hand from individual mammary glands in May 2019 (see Methods section). This sample collection led to a total of 60 clinical mastitis samples and 329 control samples, of which small aliquots (20 µl) were prepared for and analysed by LAP-MALDI MS (see Methods section for details). The control samples included 223 low-SCC (lSCC; defined as <200,000 somatic cells per ml of milk) samples and 106 high-SCC (hSCC; defined as ≥200,000 somatic cells per ml of milk) samples. The related meta-data for both control and clinical mastitis samples can be found in the Supplementary Table 1.

For model building and spectral profile classification by multivariate machine learning, lSCC and hSCC samples were considered as one control group, of which 165 samples were used for the training set and 164 were used for the test set. In each control group there were 53 hSCC samples, which led to a slightly higher percentage of hSCC samples in both the training and test sets than what is typically found in the entire herd. Two thirds of the mastitis samples were randomly assigned to the training set and one third to the test set. Using the model obtained from the training set, analysis of the test set allowed the detection of clinical mastitis with a classification accuracy of 98.9% (Figure 3a).

Pre-clinical mastitis detection

For the investigation of pre-clinical mastitis, milk sampling was undertaken in connection with the routine milking process. A volume of 2 mL of milk was taken from an automatically collected representative milk sample from each cow at the afternoon milking session weekly on the same day for a period of 24 weeks (July 2018 – December 2018), resulting in a biobank of around 12,000 milk samples. The timing of these sampling events was then compared to the

timing of the detection of clinical mastitis. From this longitudinal sample collection a total of 500 control samples and 90 mastitis samples that were collected between 0 and 7 days before the clinical event were analysed using small aliquots of 20 µl. The control samples included 364 lSCC samples and 171 hSCC samples. As before, lSCC and hSCC samples were combined and used as the control group. The related meta-data can be found in the Supplementary Table 1.

The presented model in Figure 3 was built with a training set of 313 control samples (250 lSCC and 63 hSCC samples from cows without any clinically detected mastitis within >±2 weeks of sample collection) and 8 case samples from cows that were clinically diagnosed with mastitis at the same milking session and 9 case samples collected 1 day before clinical detection, i.e. a total of 17 clinical/pre-clinical mastitis samples. The test set consisted of 102 control samples (81 lSCC and 21 hSCC samples) and 16 pre-clinical mastitis samples that were collected 2 days before clinical detection. Other analyses using training and test sets that were mixtures of mastitis samples collected at various time points up to 2 days before the clinical event showed an even higher sensitivity of up to 70%.
