# Supplementary material for: LAP-MALDI MS coupled with machine learning: an ambient mass spectrometry approach for high-throughput diagnostics
Source: Chem Sci. 2022 Jan 18;13(6):1746–58. doi: 10.1039/d1sc05171g (PMC8826629; doi:10.1039/d1sc05171g)
Supplement: SC-013-D1SC05171G-s002 [file SC-013-D1SC05171G-s002.pdf]

Electronic Supplementary Material (ESI) for Chemical Science.
This journal is © The Royal Society of Chemistry 2022

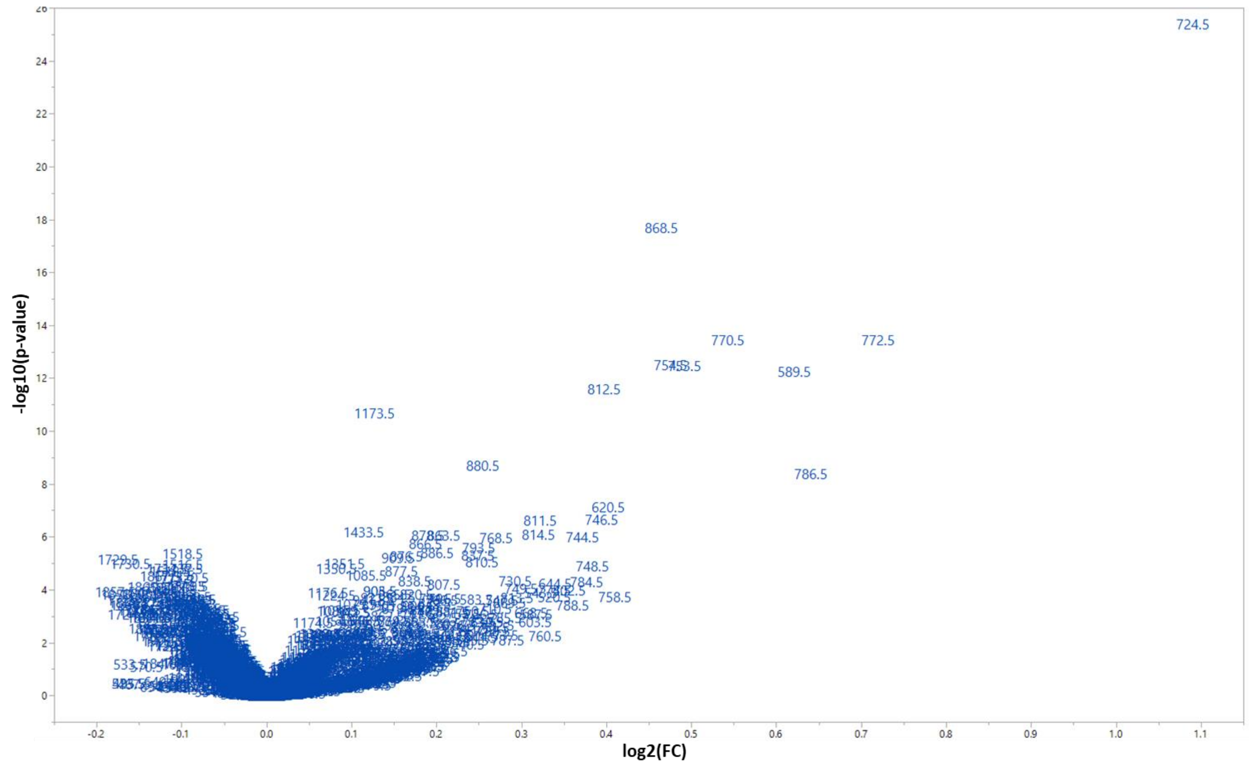

Supplementary Figure 1: Volcano plot representing the differential intensity of the mass bins as used for the pre-clinical mastitis detection. The fold change (FC) values and the p-values represent the comparisons of the relative intensities of each labelled mass bin between the control samples (lSCC and hSCC) and the pre-clinical mastitis samples collected up to 48 hours before the clinical event.

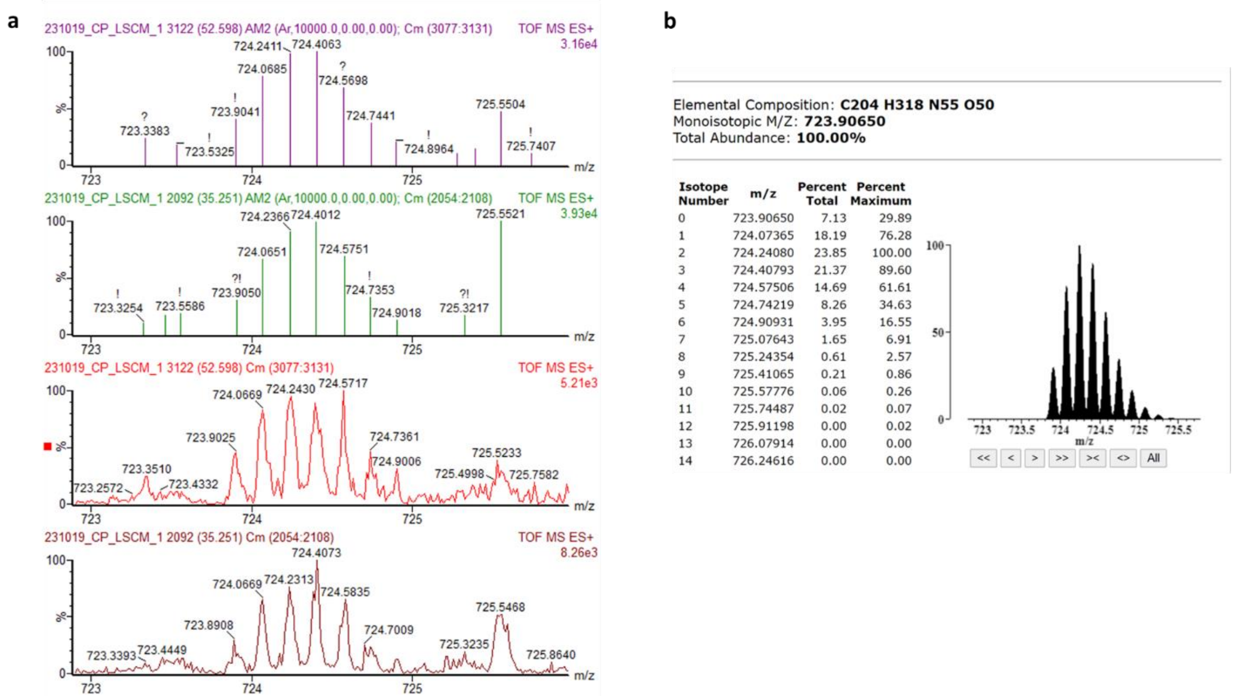

Supplementary Figure 2: The first isotopologue ion signal of the $[M+6H]^{6+}$ isracidin-containing peptide ion (RPKHPIKHQGLPQEVLNENLLRFFVAPFPEVFGKEKV; bovine alpha-S1-casein) is recorded at *m/z* 723.9 with an accuracy of <5ppm. **a.** Mass spectral raw data of two mastitis samples showing the isotopologue distribution of the $[M+6H]^{6+}$ isracidin-containing peptide ion (bottom two panels) and their corresponding centroided data (top two panels). **b.** Theoretical isotopologue distribution of the $[M+6H]^{6+}$ isracidin-containing peptide ion as obtained by the MS-Isotope function within ProteinProspector (prospector.ucsf.edu).
