# Supplementary material for: LAP-MALDI MS coupled with machine learning: an ambient mass spectrometry approach for high-throughput diagnostics
Source: Chem Sci. 2022 Jan 18;13(6):1746–58. doi: 10.1039/d1sc05171g (PMC8826629; doi:10.1039/d1sc05171g)
Supplement: SC-013-D1SC05171G-s003 [file SC-013-D1SC05171G-s003.pdf]

Electronic Supplementary Material (ESI) for Chemical Science.
This journal is © The Royal Society of Chemistry 2022

Standard Operating Procedure (SOP) for milk collection and biobanking at the University of Reading (UoR) using the animal stock and facilities at the Centre for Dairy Research (CEDAR)

Version 1.1 (07th August 2018)

Cristian Piras and Rainer Cramer
Department of Chemistry, UoR

Contents

1. Purpose 3
2. Scope 3
3. Health and Safety 3
4. Recommended equipment/material and personnel required (per collection event) 4
   4.1 Equipment 4
   4.2 Disposables 4
   4.3 Personnel required 4
5. Procedural guidelines 4
   5.1 Cryovial labelling and pre-collection preparations 5
   5.2 Cryovial sorting (first person) 6
   5.3 Sample collection (second person) 6
   5.4 Sample storage 6
6. References 8

1. Purpose

The purpose of this document is to provide guidance for the proper batch collection and storage of individual samples of dairy cow milk for further analysis by techniques such as mass spectrometry.

2. Scope

Milk represents an easy-to-collect biospecimen composed of a heterogeneous matrix of metabolites, lipids, proteins and other biomolecules (Lucey, Otter et al. 2017) that can be high importance for diagnostic purposes (Neitzel, Stamer et al. 2015, Godden, Royster et al. 2017). All these biomolecules can be subject to modifications induced by the environment and milk microbes, or in general by the abiotic and biotic conditions the sample is kept under (Wynands, LeBlanc et al. 2017). For this reason it is necessary to develop a robust sampling and storage procedure that ensures specimens minimally change or (if unavoidable) change after the collection to the same extent independent of its origin and starting composition. The latter is ideally achieved by making sure that any change will lead to an endpoint that is irreversible and quantitative.
This SOP is designed for the collection of up to 490 individual milk samples each from a different cow being milked in a milking carousel suitable for 50 dairy cows (e.g. Dairymaster 50 Rotary Milking Parlour as used at CEDAR).

Disclaimer: *This protocol has been developed and tested for use only in a research laboratory, not in a veterinary environment. No claims are made for its usefulness, accuracy or safety, and no liability can be accepted for any damages, losses or other expenses of any nature whatsoever arising from its use or supply. This protocol does not cover any legal or ethical issues.*

3. Health and Safety

Handle all milk samples as a potential source of pathogens, use the appropriate personal protective equipment and handle the samples like potential carriers of (bio)hazardous materials. Users of this protocol should have read and understood all relevant Risk and COSHH Assessments and other relevant SOPs, in particular health and safety rules and regulations and SOPs for handling farm animal body fluids, in particular raw milk samples. Before attempting to use this SOP, it is important to read and understand this document in its entirety.

4. Recommended equipment/material and personnel required (per collection event)

4.1 Equipment

- Polystyrene box (35cm*35cm*35cm) at least half full with dry ice (1)
- Black permanent markers (4)
- 10-slot cryovial racks (5)
- Printer (1)
- Rotary milking parlour (Dairymaster 50 Rotary Parlour) with 50 sampling bottles (Milk Meter Sample Bottle, 500ml, item no. 10410686/40, Milcotec)

4.2 Disposables

- 2-ml cryovials with screw cap (500 for each sampling session) (Fisherbrand™, cat n°10-500-26)
- 100-slots cryoboxes (5) (Fisherbrand™ 100-Well Microtube Storage Boxes, Product code 15579811)
- Avery No L7651 sticky labels (at least 500)
- Printing paper

4.3 Personnel required

- Two trained researchers or farm staff will be required for the whole duration of each milking/sampling session. This will be necessary in order to ensure the linear flow of sample collection (section 5.2)

5. Procedural guidelines

Always collect milk samples from the same milking session at each collection day (Antaya, Berthiaume et al. 2015). At CEDAR cows are milked twice a day and the more convenient of these two sessions is the 2:00 pm milking session. Ensure that the tasks described in section 5.1 have been finished well on time for the start of the milking session. For one person alone, preparing everything for the milking session takes around 3 hours.

Note: In order to evaluate proper sample handling and storage right from the collection point it is necessary to use an internal reference sample. For up to 25 sample collection events, collect aliquots of 2 ml of individual milk samples from 100 healthy cows each and make a pool of ~200 ml. Redistribute the obtained pool in aliquots of 1.5 ml in the same cryotubes used for all other samples and store at a -80˚ C. A total of 5 reference samples will be used for each collection event (5 cryoboxes) and will follow the entire collection and storage process from the preparation of the cryobox for sampling, to the sample harvest in the 5 different cryoboxes, their shipment and the storage of the cryoboxes in the biobank's freezer. One reference sample will be present in each cryobox.

5.1 Cryovial labelling and pre-collection preparations

5.1.1 Print all sample labels for the day and stick them on every cryovial according to the list of the cows to be sampled. See Figure 1 for the code to be used.

5.1.2 Print biobank cryobox inventory pages with samples in numerical order. See examples in Figure 1.

5.1.3 As the cows and their slot position in the milking carousel will be identified by their ID number, sort all cryovials according to their ID numbers and stick them with double sticky tape on a cow ID sorting map for easy retrieval.

5.1.4 Place the polystyrene box with dry ice at easy reach.

5.1.5 Position the clean milk collection bottles into each milk sample collector of the milking carousel.

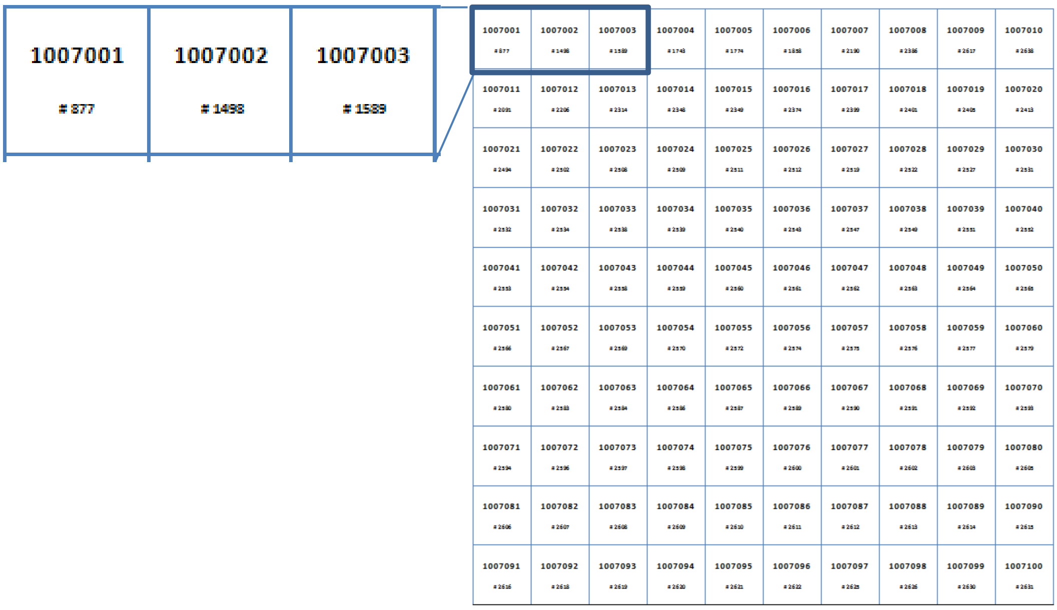

Fig. 1 Examples of biobank inventory page and labels to be printed for sorting and labeling the cryovials. The first number represents the univocal code of each sample, the second one, the code of the cow the sample has been taken from. Each cryobox will be equipped with an inventory sheet for 100 milk samples, providing the codes of all samples in the cryobox and their location within the cryobox. Assuming that the entire collection takes place within one calendar year, the following format is sufficient: MMDDCCC (MM=Month, DD=Day, CCC=sampling code for this collection day 1-490). In addition, print the cow's ID number below for additional checks and easy sample-cow association.

5.2 Cryovial sorting (first person)

5.2.1 For the first cows entering the carousel, place in order the corresponding cryovials into a 10-slot cryovial rack and label each cryovial's top with the cow's place on the carousel using the black permanent marker. If a slot on the carousel is not occupied by a cow, leave a space in the cryovial rack.

5.2.2 Hand over the fully prepared 10-slot rack to the person collecting the milk samples (see 5.3).

5.2.3 Carry on with the next 10 slots on the carousel using an empty 10-slot rack until the 50th carousel position and then start again with slot 1.

5.3 Sample collection (second person)

5.3.1 At the end of each individual milking process quickly remove the 500-ml milk collection bottle from the collector.

5.3.2 Shake it well and gently pour 2 ml of the milk in the corresponding cryovial.

5.3.3 Discard the remaining milk in the bottle by vigorously flicking it to empty the walls.

5.3.4 Refit the empty bottle at its carousel position for the next milk sampling.

5.3.5 Immediately put the freshly filled cryovial inside the polystyrene box with dry ice.

5.3.6 Repeat steps 5.3.1-5.3.5 for the next occupied carousel slot.

5.4 Sample storage

5.4.1 Transfer the whole polystyrene box to a -80˚ C freezer.

5.4.2 Prepare 5 polystyrene boxes numbered from 1 to 5 half filled with dry ice.

5.4.3 Partition samples from 1 to100 in the box number 1, samples from 101 to 200 in box number 2 and so on. The content of each polystyrene box will go into its associated cryobox.

Note: *Perform all these steps with enough dry ice per box and make sure that there is enough ventilation in the room to prevent the accumulation of high amounts of CO2 in the room.*

5.4.4 Print two different types of labels for the cryoboxes: the lateral label (Figure 2) and the lid label (Figure 1). The lateral label will be visible from the side and the lid label in the lid of the cryoboxe.

5.4.5 Place the prepared lid labels with normal tape in the inner part of the lid of each cryobox.

5.4.6 Place the cryobox number 1 in a polystyrene box half full with dry ice, close the box and wait 2 minutes for the cryobox to reach the dry ice temperature.

5.4.7 Take the polystyrene box number 1 and transfer the cryovials (1-100) into the slots of the cryobox according to the scheme in Figure 1.

5.4.8 Once finished, close the lid and place the cryobox in the – 80˚ C freezer according to the biobank map in Figure 3.

5.4.9 Repeat steps 5.4.6-5.4.8 for each of the 5 sets of samples.

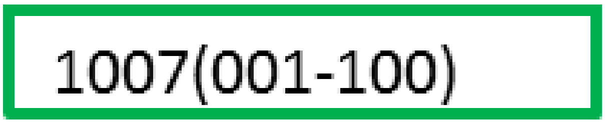

Fig. 2 Example of a lateral label for the cryoboxes lid indicating the range of samples contained in that cryobox.

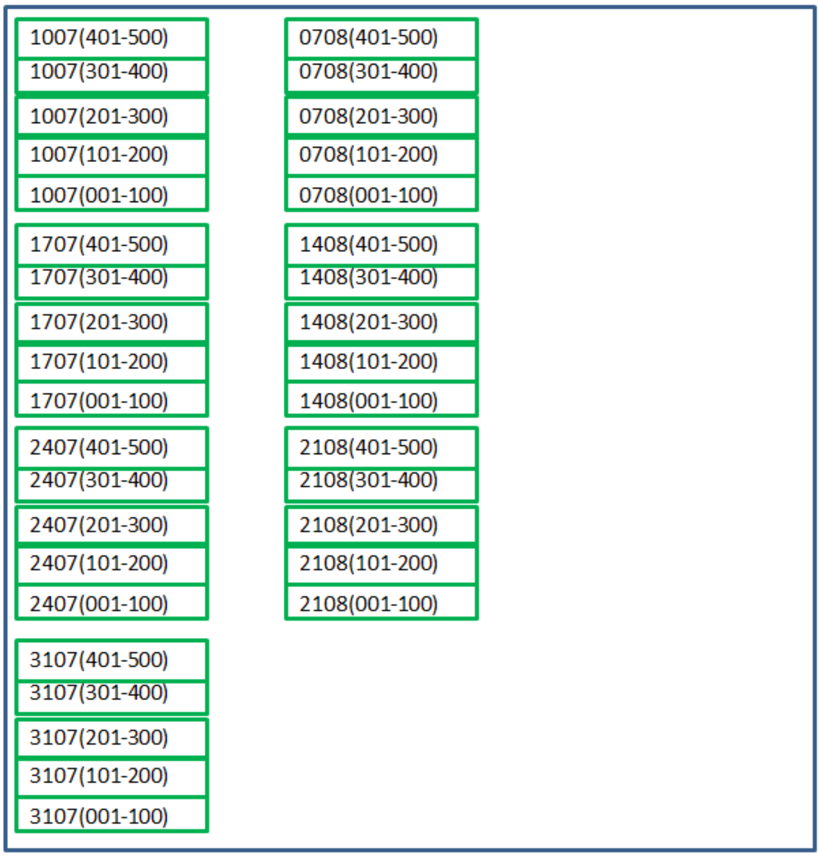

Fig. 3 Schematic representation of the storage order of cryoboxes in the biobank.

6. References

Antaya, N., et al. (2015). "Feeding red clover cut in the afternoon or morning to late-lactation dairy cows." Journal of Dairy Science **98**(10): 7335-7339.

Godden, S., et al. (2017). "Evaluation of an automated milk leukocyte differential test and the California Mastitis Test for detecting intramammary infection in early-and late-lactation quarters and cows." Journal of Dairy Dcience **100**(8): 6527-6544.

Lucey, J. A., et al. (2017). "A 100-Year Review: Progress on the chemistry of milk and its components." Journal of Dairy Science **100**(12): 9916-9932.

Neitzel, A.-C., et al. (2015). "Evaluation of different lactation curve models fitted for milk viscosity recorded by an automated on-line California Mastitis Test." Journal of Dairy Research **82**(2): 185-192.

Wynands, E., et al. (2017). "The effect of storage conditions and storage duration on milk ELISA results for pregnancy diagnosis." Journal of Dairy Science **100**(12): 9781-9786.
