# Supplementary material for: LAP-MALDI MS coupled with machine learning: an ambient mass spectrometry approach for high-throughput diagnostics
Source: Chem Sci. 2022 Jan 18;13(6):1746–58. doi: 10.1039/d1sc05171g (PMC8826629; doi:10.1039/d1sc05171g)
Supplement: SC-013-D1SC05171G-s004 [file SC-013-D1SC05171G-s004.pdf]

Electronic Supplementary Material (ESI) for Chemical Science.
This journal is © The Royal Society of Chemistry 2022

# Standard Operating Procedure (SOP) for Sample Preparation and Data Acquisition for Liquid AP-MALDI-TOF MS Profiling Analysis of Milk

## Version 1.2 (28th October 2019)

Cristian Piras and Rainer Cramer
Department of Chemistry
University of Reading

# Contents

1. Purpose ........ 3
2. Scope ........ 3
3. Health and Safety ........ 3
4. Recommended equipment/material and personnel required ........ 4
*4.1 Equipment* ........ 4
*4.2 Disposables* ........ 4
*4.3 Chemicals and solutions* ........ 4
*4.4 Personnel required* ........ 4
5. Procedural guidelines ........ 4
*5.1 Sample retrieval from the biobank (or similar storage)* ........ 5
*5.2 Defrosting and aliquoting of milk samples* ........ 5
*5.3 Defrosting and pre-processing of sample aliquots* ........ 6
*5.4 MALDI sample preparation for liquid AP-MALDI ToF MS* ........ 7
*5.5 Instrument settings, calibration, and data acquisition and analysis* ........ 8
6. References ........ 8

## 1. Purpose

The purpose of this document is to provide concise guidance and a standard operating procedure (SOP) for appropriate and analytically effective (optimised) sample preparation and data acquisition for liquid AP-MALDI-TOF MS analysis of dairy cow milk in a research lab environment.

## 2. Scope

Milk is a biofluid composed of a heterogeneous matrix of metabolites, lipids, proteins and other biomolecules (Lucey, Otter et al. 2017) and represents an easy-to-collect biospecimen that can be of high importance and great use in clinical and veterinary diagnostics (Neitzel, Stamer et al. 2015, Godden, Royster et al. 2017). For this reason, it is necessary to develop a robust procedure for sample pre-processing that will minimize the presence of pre-analytical and analytical variables that might represent the source for misleading information.
This SOP is designed for the Liquid AP-MALDI-TOF MS analysis of milk samples stored ad -80 in 2ml cryovials.

***Disclaimer:*** *This protocol has been developed and tested for research use only, not for veterinary or clinical diagnostics. No claims are made for its usefulness, accuracy or safety, and no liability can be accepted for any damages, losses or other expenses of any nature whatsoever arising from its use or supply. This protocol does not cover any legal or ethical issues.*

## 3. Health and Safety

Handle all milk samples as a potential source of pathogens, use appropriate personal protective equipment and handle samples like potential carriers of (bio)hazardous materials. Users of this protocol should have read and understood all relevant risk and COSHH assessments, in particular, health and safety rules, regulations and SOPs for handling farm biofluids such as raw milk samples.
Follow the relevant "Health and Safety Risk Assessment for Project/Activity involving Hazardous Substances" for all parts concerning the manipulation and processing of samples that involve the use of chemicals.

Before attempting to use this SOP, it is important to read and understand this document in its entirety.

## 4. Recommended equipment/material and personnel required

### *4.1 Equipment*

- -80° C freezer
- 8-channel pipettor
- CyBi™-Disk (CyBio, Analytik Jena AG, Jena, Germany) pipetting robot
- Synapt G2-Si mass spectrometer (Waters Corporation, Wilmslow, UK) modified with an in-house developed AP MALDI source as previously described (Ryumin et al. 2016)
- Hettich Centrifuge Universal 320 with microtiter plate rotor
- Standard lab vortexer

### *4.2 Disposables*

- Bel-Art™ SP Scienceware™ microcentrifuge tube racks (Fisher Scientific, Product Code.15550062)
- ABgene™ SuperPlate 96-wells PCR plates (Thermo Scientific; Fisher Scientific, Product Code.10032013)
- Axygen™ AxyMats™ sealing mat for 96-wells PCR plates (Fisher Scientific, Product Code.12577897)
- Fisherbrand™ multi-channel reservoirs (Fisher Scientific, Product Code.11334035)
- GE Healthcare Whatman™ UNIPLATE™ 96-wells clear microtiter plates (Fisher Scientific, Product Code. 11348674)
- CyBio Tips for 96-wells plates.

### *4.3 Chemicals and solutions*

- Aqueous 5% (w/v) trichloroacetic acid (TCA) solution
- Water:acetonitrile:isopropanol (1:1:1; v:v:v) solution
- Solution of 25 mg/ml α-cyano-4-hydroxycinnamic acid (CHCA) in acetonitrile/water (70:30; v:v)

### *4.4 Personnel required*

- One research scientist (+ one research assistant for sample retrieval for blinded studies)

## 5. Procedural guidelines

Milk samples will typically be obtained from frozen storage such as from the cryoboxes of the CEDAR milk biobank within Prof. Cramer's research group at the Department of Chemistry, University of Reading.

Two aliquots of 20 µl of each milk sample will be aliquoted in duplicate 96-wells PCR plates with caps and stored at -80° C until further processing.

One of the plates will undergo a protein precipitation step and subsequent MS analysis while the other one will remain stored at -80° C as backup.

***Note:*** All steps that will be performed after thawing the milk samples must be carried out by keeping the samples and extracts etc. on ice as much as possible.

### *5.1 Sample retrieval from the biobank (or similar storage)*

5.1.1 Create a written sample retrieval plan with exact sample locations based on the space available on a 96-wells microcentrifuge tube rack (or several if more than 96 samples need to be retrieved).

***Note:*** At this point consider to introduce blinded sample collection and analysis, i.e. ask a research colleague to create the retrieval plan and retrieve the samples by introducing a new sample code and randomization for the retrieved samples that is unknown to the researcher who undertakes the study until the data has been acquired and analysed.

5.1.2 Place a 96-wells microcentrifuge tube rack in a polystyrene box filled with at least 5 cm of dry ice pellets.

5.1.3 Take a section of no more than 5 cryoboxes containing around 500 samples from the -80° C freezer and place these on dry ice.

5.1.4 From each box retrieve the microcentrifuge tubes with the milk samples to be analysed and immediately place these into the 96-wells microcentrifuge tube rack. Return immediately any cryobox that is not needed anymore to the -80° C freezer.

5.1.5 Repeat until the rack is filled up according to the sample retrieval plan and store the full rack at -80° C.

5.1.6 Repeat the previous steps until all samples needed for the study have been retrieved.

### *5.2 Defrosting and aliquoting of milk samples*

5.2.1 Prepare sample processing space on ice that is sufficient for the number of samples to be processed.

5.2.2 Take the rack with the samples to be analysed out of the -80° C freezer.

5.2.3 Place the rack on a working bench to defrost at room temperature for 10 minutes.

5.2.4 Vortex the samples and place them inside the full rack on ice (+/- 0° C).

5.2.5 Keeping the samples on ice, transfer two aliquots of 20 µl from each sample tube into the wells of two different well-labelled PCR plates, thus creating duplicate plates. Make sure to be able to distinguish all duplicate plates.

5.2.6 Cover the filled PCR plates with Axygen™ AxyMats™ sealing mats for 96-wells PCR plates.

5.2.7 Store the numbered and labelled plates at -80° until further processing.

*5.3 Defrosting and pre-processing of sample aliquots*

5.3.1 Take one PCR plate with milk samples to be analysed from the freezer and thaw it at room temperature for 5 min.

5.3.2 Fill each well of a fresh 96-wells (clear) microtiter plate with 200 µl of aqueous 5% TCA solution either manually, using an 8-channel pipettor and the Fisherbrand™ multi-channel reservoirs, or with the help of a robot.

5.3.3 Place the microtiter plate with the 5% TCA solution in position 3 of the CyBi™-Disk robot.

5.3.4 Vortex the PCR plate with the samples for 5 sec per corner, centrifuge it at 500 g for 20 sec and place it in position 1 of the CyBi™-Disk robot.

5.3.5 Using the CyBi™-Disk robot and a new set of tips, pipet 100 µl of the 5% TCA solution into each well of the plate in position 1.

***Note:*** An appropriate script has to be created prior to this step. In Prof. Cramer's group this script is named "Cristian transferring and mixing 100µl" and can be found in the folder "Cristian Milk sample prep" on the computer that is controlling the CyBi™-Disk robot.

5.3.6 Remove the plate from position 1 and cover it with an Axygen™ AxyMats™ sealing mat for 96-wells PCR plates.

5.3.7 Vortex the plate for 60 sec (15 sec for each corner).

5.3.8 Centrifuge it at 2200 g for 20 min. During centrifugation fill each well of a fresh 96-wells clear microtiter plate with 200 µl of a solution consisting of water:isopropanol:acetonitrile (1:1:1; v:v:v), using an 8-channel pipettor and the Fisherbrand™ multi-channel reservoirs, or with the help of a robot, and place this plate in position 3.

5.3.9 After the centrifugation, uncover the centrifuged plate and place it in position 1.

5.3.10 Place an empty and clean plate that will be used for waste in position 8.

5.3.11 Using the CyBi™-Disk robot, carefully aspirate all of the supernatant (120 µl) and discard it in the wells of the plate in position 8, leaving the precipitated pellet intact.

***Note:*** An appropriate script has to be created prior to this step. In Prof. Cramer's group this script is named "Cristian aspirating 120µl" and can be found in the folder "Cristian Milk sample prep" on the computer that is controlling the CyBi™-Disk robot. The same tips as in step 5.3.5 can be used as long as the positions of the tips and wells are the same.

5.3.12 Using the CyBi™-Disk robot, transfer 70µl of the water:isopropanol:acetonitrile solution from the plate in position 3 to the plate in position 1.

***Note:*** An appropriate script has to be created prior to this step. In Prof. Cramer's group this script is named "Cristian transferring and mixing 80µl" and can be found in the folder "Cristian Milk sample prep" on the computer that is controlling the CyBi™-Disk robot.

5.3.13 Cover the plate in position 1 with its sealing mat, vortex it for 60 sec (15 sec for each corner), remove the sealing mat and place it back in position 1.

5.3.14 Using the CyBi™-Disk robot, aspirate all of the supernatant (70 µl) and discard it in the wells of the plate in position 8, again leaving the precipitated pellet intact.

5.3.15 Using the CyBi™-Disk robot, transfer 70µl of the water:isopropanol:acetonitrile solution from the plate in position 3 to the plate in position 1.

5.3.16 Cover the plate in position 1 with its sealing mat, sonicate it for 15 min and vortex it for 60 sec (15 sec for each corner). The applied sonication will dissolve the pellet.

5.3.17 Samples are now ready to be analysed or stored at -20° C for later analysis.

### *5.4 MALDI sample preparation for liquid AP-MALDI ToF MS*

5.4.1 Prepare the MALDI matrix solution by dissolving 25 mg of CHCA in 1 ml of acetonitrile/water (70:30; v:v) with 2-min sonication (final concentration of 25 mg/ml). Once CHCA is completely dissolved, dilute this solution 10:7 with ethylene glycol, vortex the resultant solution for 5 seconds and sonicate it for 1 minute. This prepared solution represents the liquid support matrix (LSM).

5.4.2 Pipet 20 µl of the LSM into each well of a clean 96-wells PCR plate either manually, using an 8-channel pipettor and the Fisherbrand™ multi-channel reservoirs, or with the help of a robot. Place this plate in position 3 of the CyBi™-Disk robot.

5.4.3 Place the (thawn) plate with samples to be analysed and prepared as described above in position 2 of the CyBi™-Disk robot.

5.4.4 Place a new clean 96-wells PCR plate in position 1 of the CyBi™-Disk robot.

5.4.5 Pipet with the CyBi™-Disk robot 5 µl of the milk sample solutions from position 2 into the wells of the plate in position 1.

5.4.6 Pipet with the CyBi™-Disk robot 5 µl of the LSM from the wells of the plate in position 3 into the wells of the plate in position 1. Cover the plate in position 1 with its sealing mat and vortex it for 20 sec (5 sec for each corner).

5.4.7 Spot 1.4 µl of the analyte/LSM mixtures in the wells of the plate in position 1 onto a MALDI sample plate and store the 96-wells PCR plate with the remaining analyte/LSM mixtures as a back-up or for future analysis; depending on the duration of storage either in a fridge or -80° C freezer.

5.4.8 The MALDI sample plate is ready for MS analysis but can also be stored for a few hours in a fridge.

***Note:*** Depending on the MALDI sample plate the MALDI sample spotting can be done either manually using a pipettor or with the help of a robot. If the MALDI sample plate has the microtiter plate format, it is advisable to use the CyBi™-Disk robot. Importantly, record the description of the samples and their positions on the MALDI sample plate for future reference.

### *5.5 Instrument settings, calibration, and data acquisition and analysis*

***Note:*** The following steps and instrument settings are just for general guidance for liquid AP-MALDI MS analysis of samples as prepared in the previous steps on a Waters Synapt G2-Si instrument.

5.5.1 Use the following instrument modes: MS, sensitivity, positive ion mode.

5.5.2 Calibrate the instrument with NaI over an m/z range of 100-2000.

5.5.3 Set the laser energy to approximately 10-20 µJ per single laser pulse.

***Note:*** The exact threshold laser energy that is best for an effective detection of analyte ion signals depends on the laser focus on the droplet surface and other ion source parameters (e.g. laser beam angle/position on droplet).

5.5.4 Set the automatic movement of the MALDI sample plate to 60 sec per sample with a pause of 3 sec between each sample.

5.5.5 Set the source temperature to ~80° C, the capillary to ~3.5 kV and the sampling cone to ~40 kV. Set the cone gas flow (capillary counter flow) to 150 L/h.

5.5.6 Set the detector scan time to 1 Hz and start the automatic acquisition.

***Note:*** Details about automatic data acquisition and sample stage movement depend on the exact plate size and analytes to be detected, amongst other ion source parameters and analytical objectives. Thus, no further details will be given.

Data analysis of the acquired MS profile data can be manifold and involve various software packages, in particular for classification and sample prediction models. In Prof. Cramer's group proprietary software called AMX model builder (Waters) is frequently used for this purpose.

## 6. **References**

Godden, S., et al. (2017). "Evaluation of an automated milk leukocyte differential test and the California Mastitis Test for detecting intramammary infection in early-and late-lactation quarters and cows." Journal of Dairy Science **100**(8): 6527-6544.

Lucey, J. A., et al. (2017). "A 100-Year Review: Progress on the chemistry of milk and its components." Journal of Dairy Science **100**(12): 9916-9932.

Neitzel, A.-C., et al. (2015). "Evaluation of different lactation curve models fitted for milk viscosity recorded by an automated on-line California Mastitis Test." Journal of Dairy Research **82**(2): 185-192.

Ryumin, P., et al. (2016). "Investigation and optimization of parameters affecting the multiply charged ion yield in AP-MALDI MS." Methods **104**: 11-20.
