# Supplementary material for: LAP-MALDI MS coupled with machine learning: an ambient mass spectrometry approach for high-throughput diagnostics
Source: Chem Sci. 2022 Jan 18;13(6):1746–58. doi: 10.1039/d1sc05171g (PMC8826629; doi:10.1039/d1sc05171g)
Supplement: SC-013-D1SC05171G-s006 [file SC-013-D1SC05171G-s006.pdf]

This journal is © The Royal Society of Chemistry 2022

**Cost estimation for early bovine mastitis detection using LAP-MALDI MS based on approximately 10 million samples per year (3 sec / sample; 300 plates / day)**			
	Task	Resources	Cost per sample (¢)
Staff			
	All lab work, including data provision	3x 0.5 FTE ($75,000/year)	0.75
	Sample transportation*	2x 0.3 FTE ($20,000/year)	0.2
		Subtotal	0.95
Instrumentation			
	Sample collection	Automatically by milking parlour**	0
	Robotic sample preparation	2x 96-head liquid handler platforms ($50,000; 5 years depreciation) plus service contract (10%/year)	0.3
	Other sample preparation	Various instruments (balance, sonicator, centrifuge, shaker, …; $10,000/year)	0.1
	LAP-MALDI MS analysis	1x LAP-MALDI Q-TOF instrument ($180,000; 5 years depreciation) plus service contract (10%/year)	0.54
		Subtotal	0.94
Other resources			
	Office and lab space	50sqm adequately furnished with power	0.1
	IT and communication	Data mining/dissemination ($5000/year)	0.05
	Sample collection by milking parlour	Microtiter plate and barcoding ($50/100)	0.5
	Sample transportation*	Non-staff transportation cost, including fuel ($15,000/year)	0.15
	Sample plate cleaning	Methanol / Deionised water	<0.1
	LSM bulk preparation	10uL LSM ($100/L), prepared in bulk quantities, e.g. 0.5L once a week	0.1
Analyte extraction	TCA precipitation	100uL Water (<$5/L)	<0.05
		11mg TCA ($30/kg)	0.03
		1x 100-uL pipette tip	1
	Pellet solubilisation	<100uL H_2O/IPA/ACN (<$20/L)	<0.2
		1x 100-uL pipette tip	1
MALDI sample preparation		Microtiter plate and barcoding ($50/100)	0.5
	LSM distribution	<500 tips per day	<0.02
	Analyte solution distribution	Same pipette tip used for pellet solubilisation	0
	Mixing of LSM and analyte solution	Same pipette tip used for pellet solubilisation	0
	MALDI sample spotting	1x 10-uL pipette tip	1
		Subtotal	<4.8
**Total**			**<6.69**

* Farm-site collection once daily after morning milking (up to 30,000 samples each day from approximately 10-20 local farms; total travel of max. 100-150 miles per day); ** The option to collect small amounts of milk samples on a commercial milking parlour is becoming more available. However, if this option needs to be added it is estimated that the associated capital/maintenance costs add 1-2¢ to the overall cost/sample.
